# Draft Genome Sequences of *Escherichia coli* Isolates from India

**DOI:** 10.1128/genomeA.00047-18

**Published:** 2018-02-15

**Authors:** Athira Cheruplackal Karunakaran, Arockiasamy Arun Prince Milton, Arunraj Mekhemadhom Rajendrakumar, Amit R. Sahu, A. Pandey, Sandeep Ghatak, Abhishek, Viswas Konasagara Nagaleekar, Ravikumar Gandham, Rajesh Kumar Agarwal

**Affiliations:** a Division of Veterinary Public Health, ICAR-Indian Veterinary Research Institute, Bareilly, India; b Division of Immunology, Virginia-Maryland College of Veterinary Medicine, University of Maryland, College Park, Maryland, USA; c Division of Veterinary Biotechnology, ICAR-Indian Veterinary Research Institute, Bareilly, India; d Division of Animal Health, ICAR RC for NEH Region, Umiam (Barapani), Meghalaya, India; e Division of Bacteriology and Mycology, ICAR-Indian Veterinary Research Institute, Bareilly, India

## Abstract

*Escherichia coli* causes diarrhea and extraintestinal infections in humans and animals. Here, we report the draft genome sequences of *Escherichia coli* strains 360/16 and 646, isolated from neonatal calves.

## GENOME ANNOUNCEMENT

*Escherichia coli* spp. are Gram-negative facultative anaerobic intestinal bacteria belonging to the *Enterobacteriaceae* family. Pathogenic *E. coli* can cause diarrhea and extraintestinal infections in humans and animals (1, 2). Enterohemorrhagic *E. coli* (EHEC) is responsible for outbreaks of bloody diarrhea and hemolytic-uremic syndrome (HUS) worldwide. Cattle are a natural reservoir of EHEC (3). Pathogenic *E. coli* strains cause a significant loss of neonatal animals. Understanding the genetic basis of *E. coli* has shed light on the mechanisms of pathogenesis associated with this diverse group of bacteria (4). In this study, we carried out whole-genome sequencing of two *E. coli* strains, designated 360/16 and 646, isolated from fecal samples of neonatal calves, to expand our knowledge about the genetic diversity of *E. coli* strains.

Genomic DNA was isolated from *Escherichia coli* cultures (strains 360/16 and 646), and sequence data were generated on a NextSeq Illumina platform. Two million paired-end reads for each strain were obtained upon filtering the data for quality and size using Prinseq-lite. *De novo* assembly was done using CLC Genomics Workbench version 6.0.1 and Edena version 3.13 to generate contigs. The contigs obtained from these assemblers were integrated using the Contig Integrator for Sequence Assembly (CISA). This yielded 136 and 179 contigs for *Escherichia coli* strains 360/16 and 646, respectively. These contigs were finally rearranged using Reference-Assisted Genome Ordering Utility (Ragout) version 2.0 to obtain the draft genomes of 4,748,376 bp and 5,320,632 bp for *Escherichia coli* strains 360/16 and 646, respectively. Annotation of these strains was done using the NCBI Prokaryotic Genome Annotation Pipeline. The genome of *Escherichia coli* strain 360/16 consisted of 4,649 coding sequences, 7 noncoding RNAs (ncRNAs), and 61 tRNAs, with 2 clustered regularly interspaced short palindromic repeat (CRISPR) arrays, and the genome of *Escherichia coli* strain 646 consisted of 4,901 coding sequences, 10 ncRNAs, and 58 tRNAs, with 2 CRISPR arrays.

### Accession number(s).

The genome sequences of *Escherichia coli* strains 360/16 and 646 have been deposited in the GenBank database under accession numbers CP023201 and CP023200, respectively.
